# Supplementary material for: MetaRibo-Seq measures translation in microbiomes
Source: Nat Commun. 2020 Jun 29;11:3268. doi: 10.1038/s41467-020-17081-z (PMC7324362; doi:10.1038/s41467-020-17081-z)
Supplement: Supplementary file 10 — Supplementary Data 7 [file 41467_2020_17081_MOESM10_ESM.zip › File2/Confidence_High_Taxonomy/361125_out.krona.html]

Javascript must be enabled to view this page.

members
magnitude
magnitudeUnassigned
count
unassigned
taxon
rank

361125\_out

3

2
superkingdom
3

976
phylum
3

200643
class
3

3
order
171549

171552
family
3

3
genus
838


SRS019808\_contig\_number\_19638SRS104912\_contig\_number\_13012SRS144537\_contig\_number\_51574
species
3
165179
